# Validation of the self-management ability scale (SMAS) and development and validation of a shorter scale (SMAS-S) among older patients shortly after hospitalisation

**DOI:** 10.1186/1477-7525-10-9

**Published:** 2012-01-24

**Authors:** Jane M Cramm, Mathilde MH Strating, Paul L de Vreede, Nardi Steverink, Anna P Nieboer

**Affiliations:** 1 Institute of Health Policy & Management (iBMG), Erasmus University, Rotterdam, The Netherlands; 2 Erasmus MC, Department of Public Health, Rotterdam, the Netherlands; 3 Section Health Psychology, Department of Health Sciences, University Medical Center Groningen, University of Groningen

## Abstract

**Background:**

The 30-item Self-Management Ability Scale (SMAS) measures self-management abilities (SMA). Objectives of this study were to (1) validate the SMAS among older people shortly after hospitalisation and (2) shorten the SMAS while maintaining adequate validity and reliability.

**Methods:**

Our study was conducted among older individuals (≥ 65) who had recently been discharged from a hospital. Three months after hospital admission, 296/456 patients (65% response) were interviewed in their homes. We tested the instrument by means of structural equation modelling, and examined its validity and reliability. In addition, we tested internal consistency of the SMAS and SMAS-S among a study sample of patients at risk for cardiovascular diseases.

**Results:**

After eliminating 12 items, the confirmatory factor analyses revealed good indices of fit with the resulting 18-item SMAS (SMAS-S). To estimate construct validity of the instrument, we looked at correlations between SMAS subscale scores and overall well-being scores as measured by Social Product Function (SPF-IL) and Cantril's ladder. All SMAS subscales of the original and short version significantly correlated with SPF-IL scores (all at *p *≤ 0.001) and Cantril's ladder (for the cognitive well-being subscale *p *≤ 0.01; all other subscales at *p *≤ 0.001). The findings indicated validity. Analyses of the SMAS and SMAS-S in the sample of patients at risk for cardiovascular diseases showed that both instruments are reliable.

**Conclusions:**

The psychometric properties of both the SMAS and SMAS-S are good. The SMAS-S is a promising alternate instrument to evaluate self-management abilities.

## Background

Besides a growing population of older people, a greater proportion live alone and sociological changes have forced them to rely more often on their own resources [1]. They are also hospitalised with increasing frequency as the risk for (multiple) chronic diseases increases with age [3]. They often experience losses in various domains of functioning, which leads to frailty, especially after hospitalisation [2]. Accurately assessing how they manage their well-being is thus critical.

Successful aging requires the proactive management of resources in an environment of increasing losses and declining gains [2], and depends on individuals' abilities to self-manage their lives and aging processes. Although such self-regulation is often related primarily to aspects of physical health, such as physical activity and diet [4-6], the social and psychological aspects of life - social contacts, adaptation, well-being - are equally important to older peoples' ability to 'age well' [7]. Despite acknowledgement of the importance of individuals' contributions to aging successfully and the existence of psychosocial theories of successful aging [2,8-12], relatively few suggestions have been made to help older people self-regulate and maintain their well-being [13].

The self-management of well-being (SMW) theory [13], based on the theory of social production functions (SPF) [14,15], offers guidelines for achieving better self-regulation with regard to well-being. SMW theory posits that successful aging is a life-long process of realizing and sustaining well-being, even in the face of declining resources. Rather than being the process of neutralising losses and discrepancies, successful aging focuses on individuals' reserve capacities to realize and sustain physical and social well-being using external and internal resources [13]. External resources contribute directly to aspects of well-being, such as food, shelter, fitness (physical well-being) and friends (social well-being). They tend to decline with age. Self-management abilities (internal resources) are needed to manage external resources in such a way that physical and social well-being are maintained or restored when lost [16]. SMW theory incorporates six core abilities to form the composite construct of self-management: (1) take initiatives (be instrumental or self-motivating in realizing aspects of well-being); (2) invest in resources for long-term benefits; (3) maintain variety in resources (achieve and maintain various resources for each dimension of well-being); (4) ensure resource multifunctionality (gain and maintain resources or activities that serve multiple dimensions of well-being simultaneously and in a mutually reinforcing way); (5) self-efficaciously manage resources (gain and maintain a belief in personal competence to achieve well-being); and (6) maintain a positive frame of mind. Each of these abilities must be related explicitly to the dimensions of well-being specified in the SPF theory: physical well-being (comfort and stimulation) and social well-being (affection, behavioural confirmation, and status) [13-15,17,18].

The 30-item Self-Management Ability Scale (SMAS) was developed to measure SMA [19]. Losses in functioning - something that is especially associated with hospitalisation - lead to a decreased reserve capacity for coping with losses. Self-management abilities become particularly important. Our first objective was to validate the SMAS among older people shortly after hospitalisation. The six subscales of the SMAS reflect the six SMA core abilities. Schuurmans and colleagues [19] concluded that future research could focus on shorter forms of the scale because (i) high correlations were found between some subscales and (ii) some items seemed to be less indicative of SMA (lower loadings). Our second objective was thus to reduce the number of items in the SMAS while maintaining validity and reliability.

## Methods

### Study 1

Our study was conducted in 2010 among older people who had recently been admitted to a hospital in the context of the 'Prevention and Reactivation Care Programme', which was designed to prevent loss of function in older patients due to hospitalisation and targeted older hospital patients (≥ 65 years of age) who were vulnerable to loss of function after hospital admission. Three months after hospital admission is known to be a good moment to assess effects of a programme [20,21]. Therefore, patients were interviewed three months after hospital admission. Our research is based on the pilot study of 456 patients (≥ 65 years old) prior to implementation of the 'Prevention and Reactivation Care Programme'. The results of the pilot study have been used to identify possible practical implementation problems in preparation for the main evaluation study and serve as a base for power calculations for the main study. We interviewed 296 patients in their homes (response rate 65%). This work was supported by Netherlands organisation for health research and development (ZonMw) grant number: 60-61900-98-130.

#### Ethical approval

The study protocol was approved by the medical ethics committee of the Erasmus Medical Centre, Rotterdam, the Netherlands, under protocol number MEC2011-041.

#### Measures

The 30-item SMAS consists of six five-item subscales. The scale's overall internal consistency is 0.90 [19]. Within the subscales of taking initiative, investing, self-efficacy, variety, and multifunctionality, abilities are related to the physical and social dimensions of well-being in the SPF theory [13,14]. The ability to have a positive frame of mind is considered a more general cognitive frame; its subscale is thus not directly related to specific dimensions of well-being. Average overall SMAS scores range from 5 to 30, with higher scores indicating higher SMA.

Overall subjective well-being was measured with the SPF-IL(s) (15-item Social Production Function Instrument for the Level of well-being) [17]. The scale integrates both affective and cognitive components of well-being, and measures levels of physical and social well-being. Cronbach's alpha of the SPF-IL in our study was 0.72, indicating a reliable instrument.

Cantrill's Ladder was used to assess satisfaction with life and reflects a general, cognitive evaluation of a person's overall well-being [22].

#### Analyses

The analyses included the following seven steps.

1. The sample characteristics were analysed using descriptive statistics.

2. We data-screened the items by examining the number of missing items and each item's mean and standard deviation.

3. To verify the factor structure of the questionnaire and to test whether the relationship between observed variables and their underlying latent constructs existed, confirmatory factor analysis was executed using the LISREL program version 8.80 [23]. By using structural equation modelling the overlap between items and dimensions can be traced via modification indices that were used to further refine the measurement model and eliminate potential overlap between items. No correlation errors either within or across sets of items were allowed in the model.

4. Item reduction analysis was performed to develop a short version of the questionnaire. Item removal following several criteria: (i) items were excluded following modification indices provided by LISREL and the strength of the factor loadings; (ii) item elimination stopped when the reliability of each subscale dropped below 0.65; (iii) subscales were left with as few items as possible (but a minimum of three) without loss of content and psychometric quality; and (iv) at least one physical well-being item (comfort or stimulation) and one social well-being item (affection, behavioural confirmation or status) was kept in each subscale while maintaining validity and reliability. Listwise deletion of cases with missing data on the 30 items resulted in N = 204. Imputation was done by replacing missing values with the mean of the data, restoring the original sample of N = 296.

We used four indices of model fit to test the measurement models, with cut-off criteria proposed by Hu and Bentler [24]. First, the overall test of goodness-of-fit assesses the discrepancy between the implied model and the sample covariance matrix by means of a normal-theory weighted least squares test. A plausible model has low, preferably non-significant χ^2 ^values. Chi-square is, however, overly sensitive when the sample size is large (over 200) [25], leading to difficulty in obtaining a desired non-significant level [26]. Second, the Root Means Square Error of Approximation (RMSEA) reflects the estimation error divided by the degrees of freedom as a penalty function. Values on RMSEA below 0.06 indicate small differences between the estimated and observed model. Values of up to 0.08 suggest a reasonable fit of the model in the population. Third, we used the Standardized Root Means square Residual (SRMR), which is a scale invariant index for global fit that ranges between 0 and 1. Values on SRMR lower than 0.08 indicate a good fit. Fourth, we calculated the Incremental Fit Index (IFI), which compares the independent model (i.e., observed variables are unrelated) to the estimated model. Values on IFI are preferably larger than 0.95.

5. After item reduction analyses the first full version and final short version of the instrument were tested on the non-imputed dataset (N = 204). Listwise deletion of missing data on the basis of the 18 items in the short version resulted in N = 221. We re-ran the final short version on this sample.

6. Internal consistency of the subscales was assessed by calculating Cronbach's alphas, inter-item correlations within each subscale, and correlations between subscales.

7. Validity is the degree to which a scale measures what it is intended to measure; here we focused on the construct validity of the questionnaire. Construct validity is supported if instruments purported to assess the same concept correlate substantially with one another. Since the SPF-IL and SMAS are both based on the SPF theory we evaluated construct validity by comparing the SMAS scale scores with well-being measured by the SPF-IL scale. In addition, we will compare the SMAS scale scores with well-being measured by Cantril's ladder.

### Study 2

We additionally tested the SMAS (original and short version) in another longitudinal study sample, namely patients at risk for cardiovascular diseases (low and high-risk). These patients were selected by GPs of primary healthcare practices. At both T0 and T1 Questionnaires were mailed to patients' homes. T1 was about 12 months after T0. A few weeks later, a reminder notice and another copy of the questionnaire were sent to non-respondents. Response rates were 72% (307 out of 426; T0) and 47% (200 out of 425; T1). A detailed description of the study can be found in our study protocol [27].

#### Ethical approval

The study was approved by the ethics committee of the Erasmus University Medical Centre of Rotterdam and informed consent was obtained from all participants.

#### Measures

At T0 we measured three subscales of the SMAS and SMAS-S (taking initiative, investment behavior and self-efficacy). At T1 we measured the full SMAS-S.

#### Analyses

Internal consistency of the three subscales (SMAS and SMAS-S) at T0 was assessed by calculating Cronbach's alphas. At T1 we calculated Cronbach's alphas of all six SMAS-S subscales. In addition, we assessed correlations between three subscales of the SMAS and SMAS-S at T0 and between three subscales of the SMAS-S at T0 and T1.

## Results

### Study 1

#### Sample characteristics

Respondents' median age was 75.8 (sd 6.8; range = 65-94); slightly more were female (54.2%). Just over half were married/living together (56.6%); the others were single, widowed or divorced (43.4%). Most lived independently with others (55.9%); about a third lived independently alone (37.3%); the remaining lived in elderly or nursing homes (6.8%).

#### Data screening

All items were screened for univariate and bivariate normality, and to detect outliers. Data screening information was taken into account in the stepwise procedure of the item reduction analysis. In general, the percentages of missing items were below 10%, except for item 15 (being good at certain things) of the variety subscale (table 1). This was taken into account when interpreting the results of confirmatory factor analysis.

**Table 1:** Item characteristics and factor loadings of the first full model

	Item	valid N	missing	mean	sd	λ
	*Taking Initiatives*					
1.	**How often do you take the initiative to keep yourself busy?**	**292**	**4**	**1.99**	**1.09**	**.65**
2.	How often are you engaged in making your home or room as comfortable as possible?	291	5	1.53	1.14	.46
3.	**How often do you take the initiative to get in touch with people who are dear to you?**	**291**	**5**	**1.93**	**1.05**	**.82**
4.	Do you sometimes try to be good at something?	283	13	1.67	1.22	.48
5.	**How often do you make an effort to have friendly contacts with other people?**	**291**	**5**	**1.67**	**1.06**	**.80**
	*Investment Behavior*					
6.	**Do you ensure that you have enough interests on a regular basis (such as a hobby) to keep you active?**	**291**	**5**	**2.16**	**1.16**	**.65**
7.	Do you make sure that you get enough physical exercise in order to stay fit longer?	292	4	1.72	1.18	.49
8.	Do you occasionally do something so that your contact with your acquaintances remains good?	288	8	1.47	.98	.64
9.	**Do you devote some time and attention to those who are dear to you in order to maintain good contact?**	**288**	**8**	**2.02**	**1.00**	**.77**
10.	**Do you keep busy with the things you are good at so that you stay good at them?**	**285**	**11**	**1.81**	**1.21**	**.66**
	*Variety*					
11.	**How many hobbies or activities do you have on a regular basis?**	**289**	**7**	**2.03**	**1.18**	**.67**
12.	Do you have different ways to relax when necessary?	289	7	.62	.82	.34
13.	**Do you have different occasions on which you have friendly contacts with others?**	**286**	**10**	**2.26**	**1.27**	**.73**
14.	With how many people do you have a confidential relationship?	285	11	2.54	1.32	.43
15.	**Are there certain things that you are good at?**	**266**	**30**	**1.41**	**1.26**	**.61**
	*Multifunctionality*					
16.	**The activities I enjoy, I do together with others**.	**290**	**6**	**1.60**	**1.15**	**.59**
17.	**I sometimes help the people I care about**.	**285**	**11**	**1.82**	**1.08**	**.75**
18.	**Others benefit from the things I do for my pleasure**.	**277**	**19**	**1.66**	**1.04**	**.74**
19.	I generally spend my holidays with others.	289	7	2.68	1.39	.32
20.	I practice my hobbies together with others.	288	8	1.29	1.17	.45
	*Self-efficacy*					
21.	**Are you able to find agreeable activities?**	**288**	**8**	**2.18**	**1.03**	**.77**
22.	Are you capable of taking good care of yourself?	287	9	2.74	1.07	.56
23.	**Are you able to have friendly contacts with others?**	**290**	**6**	**2.23**	**1.10**	**.86**
24.	**Are you able to let others know that you care about them?**	**286**	**10**	**2.24**	**1.01**	**.67**
25.	Are you good at something?	282	14	1.63	1.13	.55
	*Positive Frame of Mind*					
26.	How often are you able to see the positive side of the situation when something disagreeable happens?	278	18	1.89	1.25	.63
27.	**When things go against you, how often do you think that it could always be worse?**	**280**	**16**	**2.22**	**1.34**	**.79**
28.	When you are not doing well, how often do you think that there are others who are worse off?	276	20	2.21	1.30	.76
29.	**When you have a bad day, how often do you think that things will be better tomorrow?**	**275**	**21**	**2.37**	**1.27**	**.71**
30.	**When things are not going so well, how often do you succeed in thinking positively?**	**284**	**12**	**2.37**	**1.13**	**.72**

Items in bold are included in the short version

#### Confirmatory Factor Analysis

All items (table 1) had factor loadings above 0.40 on the intended factor except item 12 (having different ways to relax) and item 18 (doing things for pleasure that benefit others), which were 0.34 and 0.31 respectively. Each SMA measure (except positive frame of mind) was designed with regard to the five dimensions of well-being. We tested the matrix model where each SMA is linked to the dimensions of well-being. The indices in table 2 clearly showed a good fit: a relatively small χ^2^; SRMR had small residuals, indicating good global fit; a small RMSEA within its 90% confidence interval; and a large IFI indicating a good model. Although significant, the Normal Theory Weighted Least Square χ^2 ^statistic is not surprising given its sensitivity to sample size. Together the analyses showed that the underlying factors of the items were indeed the dimensions of abilities and well-being. A one-factor model without distinguishing the six subscales resulted in a worse fit (χ^2 ^= 2394.115 (*p *≤ 0.0); RMSEA 0.0978; IFI 0.909; SRMR 0.0939).

**Table 2:** Model fit indices

*full model with 6 abilities systematically linked to dimensions of well-being as latent variables*				
**On imputed data (n = 296)**	Χ^2 ^(p)	RMSEA	IFI	SRMR
30 items	837.874 (0.0)	.0438	.985	.0568
**Listwise deletion 30 items (n = 204)**				
30 items	740.991 (0.0)	.0472	.984	.0603
***Full and short models with 6 abilities not systematically linked to dimensions of well-being as latent variables***				
**On imputed data (n = 296)**				
30 items	1507.845 (0.0)	.0689	.957	.0718
Final short version 18 items	523.786 (0.0)	.0740	.971	.0644
**Listwise deletion 30 items (n = 204)**				
30 items	1274.298 (0.0)	.0734	.955	.0804
Final short version 18 items	454.335 (0.0)	.0807	.967	.0755
**Listwise deletion 18 items (n = 221)**				
Final short version 18 items	501.856 (0.0)	.0845	.964	.0742

If we a priori do not link each measure of SMA to the five dimensions of well-being the indices of model fit also showed that the model fit was sufficient (table 2). The RMSEA was just above cut-off value, indicating reasonable fit. IFI value was 0.955, near the cut-off value of .95, and SRMR was well below the cut-off value of 0.08. All indices indicated that the model not systematically linked to the five dimensions of well-being was acceptable, but left room for improvement.

#### Item reduction analysis

Following the factor loadings, modification indices, and an internal consistency check of each subscale, the stepwise procedure resulted in the elimination of 12 items. With respect to the 'investment behavior' subscale, modification indices and factor loadings showed that item 7 (getting enough exercise) could be eliminated. The results on the other items of the subscale showed some contradictory results. Eliminating item 6 (having a hobby) resulted in a better fit of the model; however, the physical component was no longer represented in the remaining items (8, 9 and 10) and led to a Cronbach's alpha below 0.70. Therefore, based on a lower factor loading of item 8 and construct validity, item 6 remained in the selection and item 8 (actively maintain contact with acquaintances) was eliminated.

The final short version consisted of 18 items with three items for each subscale (table 1). Item reduction was possible without loss of model fit; in fact, its overall fit was better than the full version. Due to a decrease in the number of estimated parameters, the Normal Theory Weighted Least Square χ^2 ^significantly decreased to 530.427. RMSEA still indicated reasonable fit. The value of IFI improved to 0.967, indicating that the specified relations between variables were well supported by the data. The SRMR index decreased to 0.0669, still considerably below the cut-off point of 0.08, indicating good global fit. The final short model on imputed data resulted in comparable factor loadings. A re-run of the full model and item reduction analysis on the non-imputed dataset (N = 217) resulted in somewhat less favourable but still acceptable fit indices and comparable factor loadings.

#### Internal consistency and inter-correlations

Internal consistency as represented by Cronbach's alpha ranged from sufficient for the 'variety' and 'multifunctionality' subscales to very good for the 'taking initiative' subscale (table 3). The correlations between the full original subscales and short subscales were also good (0.90-0.95) indicating acceptable coverage of the original sub-dimensions. The six subscales were significantly and positively correlated, indicating conceptually related subscales. A one-factor model without distinguishing the six subscales resulted in a worse fit (χ^2 ^= 977.270 (*p *≤ 0.0); RMSEA 0.109; IFI 0.929; SRMR 0.0900). In addition, factor loadings were high on the six dimensions, which indicates that although the SMAS-S subscales are related they do represent separate concepts.

**Table 3:** Scale characteristics and inter-correlations of the shortened subscales (n = 296)

	items short version	Cronbach's alpha	original full scale	scale mean (sd)	inter-item correlations range	1	2	3	4	5
1. Taking Initiatives	1, 3, 5	.77	.91*	1.86 (.88)	.43-.70					
2. Investment Behavior	6, 9, 10	.71	.93*	1.98 (.90)	.42-.50	.62*				
3. Variety	11, 13, 15	.69	.93*	1.90 (.97)	.38-.50	.47*	.53*			
4. Multifunctionality	16, 17, 18	.69	.90*	1.70 (.86)	.34-.62	.43*	.61*	.53*		
5. Self-efficacy	21, 23, 24	.77	.94*	2.22 (.87)	.47-.61	.63*	.71*	.49*	.57*	
6. Positive Frame of Mind	27, 29, 30	.74	.95	2.31 (1.01)	.48-.50	.33*	.40*	.19*	.22*	.51*

* p < 0.01

#### Validity

To estimate construct validity of the instrument, we looked at correlations between SMAS subscale scores and overall well-being scores. All SMAS subscales of the original and short versions significantly correlated with SPF-IL scores (all at *p *≤ 0.001) and Cantril's ladder (for cognitive well-being *p *≤ 0.01; all other subscales *p *≤ 0.001), indicating convergent validity. The relative strength of association with SPF-IL scores are the same for the original SMAS (range = 0.311-0.593) and the short version (0.311-0.580), which also applies to the association between Cantril's ladder and SMAS (0.155-0.430) and SMAS-S (0.150-0.420).

### Study 2

We additionally tested the SMAS and SMAS-S in another study sample, namely patients at risk for cardiovascular diseases (low and high-risk). At T0 respondents' median age was 59.8 (sd 9.6; range = 31-88); slightly more were female (56.4%). The majority were married/living together (76.6%). At T1 respondents' median age was 60.2 (sd 9.1; range = 34-86); 58.2% female and 79.1% were married/living together.

At T0 we tested the three subscales 'taking initiative', 'investment behavior' and 'self-efficacy' of both the SMAS and SMAS-S for internal consistency. Cronbach's alpha of the SMAS and SMAS-S were both reliable: 'taking initiative' (0.79 SMAS *vs *0.78 SMAS-S), 'investment behavior' (0.83 SMAS *vs *0.78 SMAS-S), and 'self-efficacy' (0.84 SMAS *vs *0.80 SMAS-S). At T1 we tested all six subscales of the SMAS-S. These results showed that the SMAS-S is a reliable instrument (range from 0.73 for 'positive frame of mind' to 0.85 for 'self-efficacy'). The correlations between the three original SMAS subscales and short subscales (SMAS-S) at T0 were also good (0.93-0.95) indicating acceptable coverage of the original sub-dimensions. The three SMAS-S subscales measured at T0 and T1 were also significantly related (0.57-0.70) indicating reliability.

## Discussion

Due to high risk of functional losses among older people after hospitalisation, SMA becomes particularly important. Our objectives were to (1) validate the SMAS among older people who had recently been admitted to a hospital and (2) reduce the number of items in the SMAS while maintaining validity and reliability. After performing an item reduction analysis, the resulting 18-item short version (SMAS-S) was shown to be reliable and valid. The results of the confirmatory factor analyses revealed good indices of fit with the SMAS and SMAS-S. The SMAS-S is thus a good alternative to the lengthier SMAS. We also found high correlations between some subscales in the SMAS and several items may have been less indicative of SMA (lower loadings). Our study showed that the subscales of the SMAS-S represented separate concepts. Therefore, SMA may even be better measured using the SMAS-S. Each measure of 30-item SMAS (except positive frame of mind) is however, specifically related to the five dimensions of well-being specified in the SPF theory and thus provides insight into all five well-being dimensions; the SMAS-S items are related to the two higher-level dimensions (physical and social well-being).

We found support for convergent validity of the original SMAS and SMAS-S through high correlations between the SMA dimensions and subjective well-being as measured by SPF-IL and Cantril's ladder.

We could not evaluate several psychometric properties in this study: the relationship of the SMAS-S with other self-management instruments, assessment of the SMAS-S responsiveness, its predictive value (e.g., clinical outcomes), and different modes of administration. They thus remain undefined. The instrument's sensitivity to change requires further investigation. We recommend testing the English version of the SMAS-S in other countries to ensure international validity. Last, our sample size was relatively small and our sample population was older people who had recently been discharged from the hospital. Future research is necessary to test the SMAS-S on other as well as larger populations. While the SMAS is validated and designed to assess self-management abilities among older people, this study additionally tested the SMAS-S among patients at risk for cardiovascular diseases (aged 30+). Our study findings show promising results to assess self-management abilities with the SMAS-S among other populations.

## Conclusion

We conclude that the psychometric properties of both the SMAS and SMAS-S are good and the subscales of SMAS-S clearly represent separate concepts. The SMAS-S is a promising alternate instrument to evaluate self-management abilities. Having a shorter instrument makes it more feasible to assess SMA in a broader number of people, especially among frail older populations.

## Competing interests

The authors declare that they have no competing interests.

## Authors' contributions

JC Preparation of the paper; analyses and interpretation of data; final approval of the version to be published. MS Preparation of the paper; analyses and interpretation of data; final approval of the version to be published. PV Preparation of the paper; acquisition of subjects and data; final approval of the version to be published. NS Preparation of the paper; analyses and interpretation of data; final approval of the version to be published. AN Acquisition of subjects and data; study concept and design; preparation of the paper; analyses and interpretation of data; final approval of the version to be published.
